# Long-term health-related quality-of-life and psychosocial outcomes after uterus transplantation: a 5-year follow-up of donors and recipients

**DOI:** 10.1093/humrep/dead245

**Published:** 2023-11-23

**Authors:** Stina Järvholm, Alva Kättström, Niclas Kvarnström, Pernilla Dahm-Kähler, Mats Brännström

**Affiliations:** Department of Obstetrics & Gynecology, Institue of Clinical Sciences, Sahlgrenska Academy, University of Gothenburg, Göteborg, Sweden; Department of Obstetrics & Gynecology, Institue of Clinical Sciences, Sahlgrenska Academy, University of Gothenburg, Göteborg, Sweden; Transplantation, Institute of Clinical Sciences, Sahlgrenska Academy, University of Gothenburg, Göteborg, Sweden; Department of Obstetrics & Gynecology, Institue of Clinical Sciences, Sahlgrenska Academy, University of Gothenburg, Göteborg, Sweden; Department of Obstetrics & Gynecology, Institue of Clinical Sciences, Sahlgrenska Academy, University of Gothenburg, Göteborg, Sweden; Stockholm IVF-EUGIN, Stockholm, Sweden

**Keywords:** infertility, psychology, quality-of-life, transplantation, uterus

## Abstract

**STUDY QUESTION:**

What are the outcomes regarding health-related quality-of-life, mood, and marital relationship of recipients and donors 5 years after uterus transplantation (UTx) and uterus donation?

**SUMMARY ANSWER:**

Both recipients and donors generally demonstrated long-term stability regarding psychosocial outcomes but with negative deviations associated with unsuccessful outcomes.

**WHAT IS KNOWN ALREADY:**

UTx is the first infertility treatment for women with absolute uterine factor infertility. The procedure can be performed with either a uterus donation from a live donor (LD), typically a close relative, or from a deceased, multi-organ donor. There are many potential stressful events over several years after UTx both for recipients and for LDs and these events may have impacts on quality-of-life and mental well-being.

**STUDY DESIGN, SIZE, DURATION:**

This, prospective observational cohort study includes the nine recipients and LDs of the first human UTx trial. They were assessed in 2017–2018 by questionnaires 5 years after UTx.

**PARTICIPANTS/MATERIALS, SETTING, METHODS:**

The nine recipients (ages 32–43 years) and their respective LDs (ages 44–67 years) were either related (n = 8) or friends (n = 1). Eight recipients had congenital uterine absence and one was hysterectomized due to cervical cancer. For two recipients, UTx resulted in early graft failures, while six of the other seven recipients gave birth to a total of eight babies over the following 5 years. Physical and mental component summaries of health-related quality-of-life were measured with the SF-36 questionnaire. Mood was assessed by the Hospital Anxiety and Depression Scale. Relationship with partner was measured with the Dyadic Adjustment Scale. Comparisons were made between the values after 5 years and the values before uterus donation/transplantation.

**MAIN RESULTS AND THE ROLE OF CHANCE:**

Five years after primary UTx, the majority of recipients scored above the predicted value of the general population on quality-of-life, except for two women, one of whom had a viable graft but no live birth and one recipient who was strained by quality-of-life changes, possibly related to parenthood transitions. Regarding mood, only one value (anxiety) was above the threshold for further clinical assessment. Recipients showed declining satisfaction with their marital relationships, but all reported scores above the ‘at risk for divorce’ threshold at the time of the final assessment in our study. The LDs were all found to be stable and above the predicted value of the general population regarding mental components of quality-of-life. Three LDs showed declined physical components, possibly related to older age. Only one LD reported a value in mood (anxiety) that would need further assessment. The marital satisfaction of LDs remained stable and unchanged compared to baseline values. Notably, the two recipients with early graft failures, and their related LDs, regained their mental well-being during the first years after graft failure and remained stable after 5 years.

**LIMITATIONS, REASONS FOR CAUTION:**

The restricted sample size and the single-centre study-design are limitations of this study. Additionally the study was limited to LD UTx, as opposed to deceased donor UTx.

**WIDER IMPLICATIONS OF THE FINDINGS:**

Our study shows that both LDs and recipients had acceptable or favourable quality-of-life outcomes, including mood assessment, at the 5-year follow-up mark, and that failure to achieve a live birth negatively affected these modalities both for LDs and recipients. Moreover, an important finding was that LDs and recipients are not reacting with depression after hysterectomy, which is common after hysterectomy in the general population.

**STUDY FUNDING/COMPETING INTEREST(S):**

Funding was provided by the Jane and Dan Olsson Foundation for Science, Knut and Alice Wallenberg Foundation, Handlanden Hjalmar Svensson Foundation, Swedish Governmental ALF Grant, and Swedish Research Council. There are no conflicts of interest to disclose.

**TRIAL REGISTRATION NUMBER:**

NCT01844362.

## Introduction

Uterus transplantation (UTx) was first attempted in year 2000 in the single case of a live donor (LD) UTx. The procedure however resulted in a graft failure 3 months after UTx (Fageeh *et al.*, 2002). In 2012, we initiated the first human clinical UTx trial, including nine LD UTx procedures (Brännström *et al.*, 2014). This trial resulted in the world’s first live birth after UTx in 2014 (Brännström *et al.*, 2015) and in eight subsequent births within the interval from 2 to 6 years after UTx (Brännström *et al.*, 2022). The reproductive lives of the participants were completed by graft removals by hysterectomy at different time points and on disparate indications after UTx. Today, more than 40 live births from more than 70 UTx procedures with donations from both LDs and from deceased donors (DDs) have been reported (Brännström *et al.*, 2023a).

UTx is, unlike most other organ transplantations, not a lifesaving intervention but a quality-of-life enhancing type of transplantation. Importantly, it is the first available treatment for absolute uterine factor infertility (AUFI), which affects around 20 000 women of fertile age in a total population of 100 million (Sieunarine *et al.*, 2005; Milliez, 2009). The great majority of UTx procedures performed and reported into the international UTx registry (Brännström *et al.*, 2023b) have involved transplantations in women with Mayer–Rokitansky–Küster–Hauser syndrome (MRKHs), which is characterized by congenital absence of the uterus and the upper part of the vagina (Herlin *et al.*, 2020). Receiving the diagnosis of MRKHs may negatively affect the psychological status of the woman, who typically is diagnosed during the sensitive adolescence period (Heller-Boersma *et al.*, 2009). Even though most women with MRKHs are, later in life, almost comparable to the general population regarding quality-of-life, aspects of fertility, and sexual self-esteem are reported to stay negatively affected (Weijenborg *et al.*, 2019).

Around three quarters of all UTx procedures which have been performed worldwide are LD UTx even though it involves more complex donor surgery and a risk for the donor, as compared to the use of DD (Brännström *et al.*, 2023a). The concerns of LDs about their hysterectomy surgery and, later, about the reproductive outcome and health of the respective recipient, who in most cases is closely related to the LD, may also affect their psychological health and quality-of-life of the LDs. The need for psychological follow-up of both recipients and LDs was pointed out in a position paper from several UTx centres (Allyse *et al.*, 2018).

After UTx, birth of at least one child is the goal and it may take several years from UTx until this is achieved. Therefore, psychosocial follow-up over several years is crucial in evaluating the safety and outcome of the UTx. Comprehensive quantitative data regarding the health-related psychosocial well-being of both recipients and LDs have been reported in detail for 2 and 3 years after UTx (Järvholm *et al.*, 2019, 2020). Moreover, we have presented limited data of health-related psychosocial well-being in relation to reproductive outcomes at the 4-year time point after surgery (Brännström *et al.*, 2022). Recipients were psychologically stable over the period of the studies, but strains were reported soon after the transplantation surgery (Järvholm *et al.*, 2015) and during the period when pregnancy was attempted and/or when outcomes were unsuccessful (Järvholm *et al.*, 2020). Partners’ responses showed a similar pattern, with general stability during the initial post-UTx period trial but they too were negatively affected when outcomes were unsuccessful. A decline in satisfaction with marital relationship was seen among the partners regardless of outcome, however the results were still above the clinical threshold for distress (Järvholm *et al.*, 2020). The psychosocial health of LDs was generally stable both before transplantation and after 3 years of follow-up year three. Declines were reported and may have been associated both with unsuccessful outcomes of the graft as well with general life changes, such as retirement (Järvholm *et al.*, 2019). Qualitative follow-up studies showed that the state of having a uterus affected the recipient’s self-image in a positive way, but this was also associated with strains regarding body image and sexuality, and after giving birth the recipients saw themselves as almost like everyone else but with a special experience in their road to motherhood (Järvholm *et al.*, 2020, 2022). From a large UTx centre in USA, a 2-year follow-up study with qualitative data of recipients reported positive impacts regarding their reproductive autonomy and female identity (Wall *et al.*, 2022).

Herein, we report the 5-year outcomes of the first human series of UTx recipients and donors concerning health-related quality-of-life, mood and marital relationship. To our knowledge, this is the longest follow-up of women undergoing uterus donation and transplantation.

## Materials and methods

### Participants

The participants represent the cohort of the nine recipients and donors participating in the first human clinical LD UTx trial that took part in Gothenburg, Sweden, with surgeries performed in 2012–2013. The recipients had different outcomes during the period from UTx to follow-up after 5 years. Patients 2 and 9 had early graft failures after three and a half months and 3 days, respectively (Brännström *et al.*, 2014). During the period of the study (up to 60 months after transplantation), six recipients delivered healthy children (Brännström *et al.*, 2022). The deliveries were from Recipients 5, 7, 1, 8, 3, and 6, with their first deliveries taking place 19, 20, 26, 28, 35, and 53 months post-UTx, respectively (Mölne *et al.*, 2017). Two recipients had second deliveries during the 5-year period (Patient 1 at 52 months, and Patient 3 at 52 months, post-UTx). Two recipients and one LD separated from their partners during the study period. The ages at the 5-year follow-up were 32–43 years for recipients and 44–67 years for LDs.

### Ethical approval

The study (ClinicalTrials.gov NCT01844362) was approved by the Regional Ethics Committee, University of Gothenburg, Sweden, in May 2012. Written informed consent to participate in the study was obtained independently from recipients and LDs prior to enrolment, which was typically approximately 1 year prior to the UTx procedure.

### Procedure

The LDs and recipients were assessed by independent psychologists at inclusion to uncover and minimize the effects of inherent coercion. Every year after UTx, up to 5 years after, the participants received a booklet by post with questionnaires. The completed questionnaires were returned in prepaid envelopes. Patients who did not return the questionnaires received a reminder by post or phone on two occasions. Despite this, two recipients (Patients 4 and 7) and one LD (Patient 8) did not fill in the questionnaires for Year 5 and, accordingly, their year four values were used in the group analysis. Additionally, the recipients and LDs were followed-up by the psychologists on request.

### Instruments

The questionnaires used (see below) were chosen since they are well established and validated research tools. Notably, given the novelty of the UTx field, none of the instruments have yet been validated specifically for the UTx group. Data from Years 2, 3, and 4 are given in previous publications (Järvholm *et al.*, 2019, 2020; Brännström *et al.*, 2022).

#### Health-related quality-of-life

Health-related quality-of-life was measured by SF-36, a well-established validated questionnaire in medical research (Fayers and Machin, 2013). The questionnaire assesses eight health domains of both mental and physical health, by 36 questions. The physical component summary (PCS) consists of the subscales physical functioning, role limitations due to physical health, bodily pain, and general health. Similarly, the mental component summary (MCS) consists of subscales measuring vitality, social function, and role-limitations due to emotional problems (Ware, 2000). When calculating the summary scores for the MCS and for the PCS, normative values for a Swedish population were used. Mean scores for a Swedish population were both 50, with a SD of 10 (Ware *et al.*, 1998).

#### Mood

Depression and anxiety were evaluated with the Swedish version of Hospital Anxiety and Depression Scale (HADS) (Zigmond and Snaith, 1983). This scale was developed to screen for depression and anxiety among patients in non-psychiatric hospital clinics and includes 14 items (7 for anxiety, 7 for depression). The items are scored on a 4-point Likert scale (scores from 3 to 0) from ‘Yes definitely’ to ‘No, not at all’. A systematic review with a large number of studies (Bjelland *et al.*, 2002) used a threshold of 8/21 and higher to identify either anxiety (A) or depression (D), and accordingly the need for further clinical assessment. When tested on a large Swedish non-clinical population of women, mean scores of HADSA and HADSD were 4.76/21 and 3.76/21, respectively (Lisspers *et al.*, 1997).

#### Marital relationship

In order to measure satisfaction with the quality of the LDs’ and recipients’ relationships, the Swedish version of the Dyadic Adjustment Scale (DAS) was used (Hansson, 1994). DAS consists of 32 items, which are either Likert-type (0–5) or dichotomous (yes/no) with a maximum score of 151 (higher scores indicate better quality of the relationship). The questionnaire reflects levels of agreement within the couple and discriminates between well-adjusted couples, poorly adjusted couples, and couples with a high likelihood of separation. A value of 100 is used as a cut-off to discriminate between non-distressed and distressed couples (Spanier, 1976). A later study (Graham *et al.*, 2006) suggested that a continuum between 92 and 107, rather than a sharp cut-off at 100, should be used. When assessing the marital relationship, the mean score for DAS in a non-clinical Swedish cohort of females was 118.3/151 (Lundblad and Hansson, 2005).

### Statistics

Results of SF-36, HADS, and DAS are presented as descriptive statistics.

## Results

### Health-related quality-of-life

**Figure 1.**
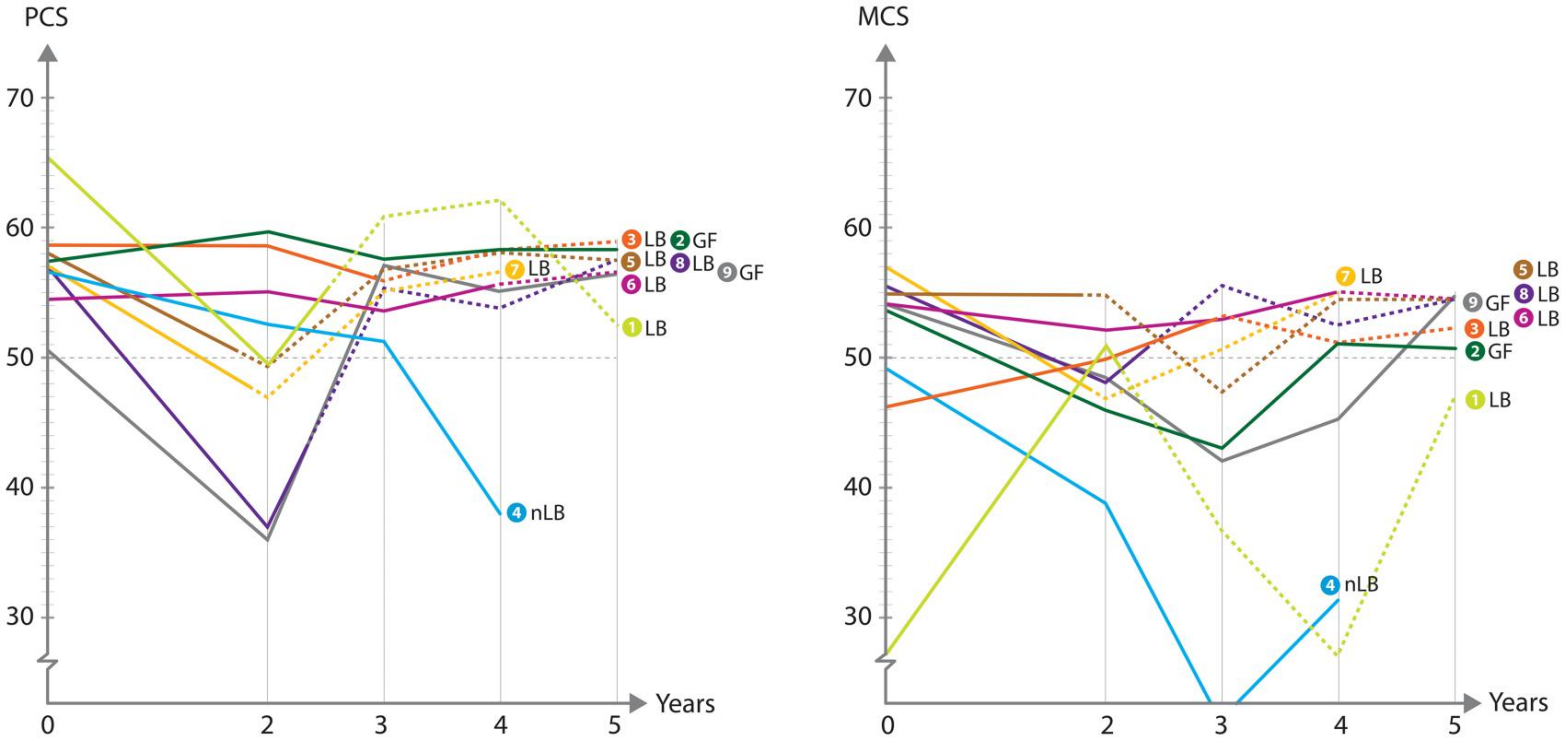
**Individual recipient-summarized scores on the physical component summary (PCS) and the mental component summary (MCS) of SF-36, up to 5 years after transplantation.** LB, live birth; nLB, no live birth; GF, graft failure.

The recipients’ individual scores of SF-36, concerning PCS and MCS, are shown in Fig. 1. The 5-year values of the PCS among the recipients were relatively similar, with all but one above the predicted value of the general population (50) and comparable to or above their values at baseline, before UTx. Recipient 1, with her first live birth 26 months after UTx, delivered her second child 52 months after UTx. She showed a clear decline of 13 points from baseline value (before UTx) at the 2-year follow-up but scored above the mean value at follow-up 5 years after UTx. Recipient 4, with no live birth and six miscarriages (Brännström *et al.*, 2022) during the study period, showed a continuous decline of PCS during the study period, with the last value being 38 (Year 4) (Fig. 1).

Regarding MCS, a similar pattern to PCS was seen in the majority of women, with values above the predicted value of the general population (50) in seven women at Year 5, and decreased values in two recipients. Recipient 1 showed large fluctuations in scores with a low MCS at start and values just below the threshold at Year 5. Recipient 4 scored normal values before UTx, but had decreasing scores over the entire study period.

**Figure 2.**
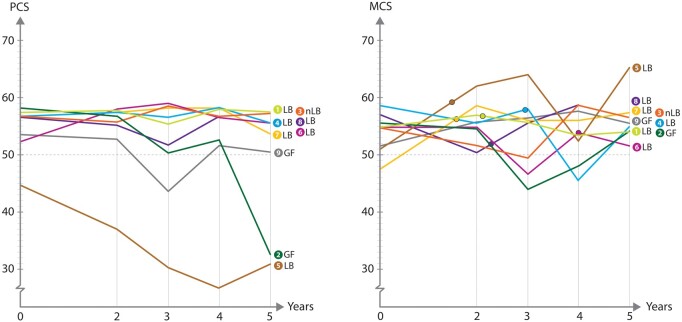
**Individual live donor-summarized scores on the physical component summary (PCS) and the mental component summary (MCS) of SF-36, up to 5 years after transplantation.** LB, live birth; nLB, no live birth; GF, graft failure.

The individual values of SF-36 for the LDs are shown in Fig. 2. Concerning six of the LDs, their PCS was stable during the study period. A decline from baseline values was seen in three LDs. These LDs were all above 60 years of age at uterus donation and reported possibly age-related deteriorations in physical ability during the follow-up period, being above 65 years of age at data-recording 5 years after the donor hysterectomy.

Regardless of outcome of the donated graft (live birth/no live birth/graft failure) their MCS at Year 5 were normal and similar to baseline for all LDs. The medians and ranges for SF-36 are presented in Table 1.

**Table 1.** Values on quality-of-life (SF-36: physical component summary (PCS) and mental component summary (MCS)), anxiety (HADSA), depression (HADSD), and marital satisfaction (DAS) for uterine transplantation recipients and donors at inclusion and at Year 5 follow-up.

	Recipients* n = 9^1^	Donors* n = 9^1^
**SF-36 PCS at inclusion**	57.1 (50.7–85.3)	56.7 (24.7–58.6)
**SF-36 PCS Year 5**	56.9 (38.1–59.0)	55.6 (30.9–57.5)
**SF-36 MCS at inclusion**	54.1 (19.7–57.0)	53.9 (45.4–58.6)
**SF-36 MCS Year 5**	54.4 (31.4–55.0)	55.5 (51.8–65.3)
**HADSA at inclusion**	3 (0–12)	5 (0–12)
**HADSA Year 5**	1 (0–13)	1 (0–8)
**HADSD at inclusion**	0 (0–3)	0 (0–4)
**HADSD Year 5**	0 (0–8)	1 (0–4)
**DAS at inclusion**	131 (112–142)	130 (112–151)
**DAS Year 5^2^**	115 (105–131)	132 (105–144)

* Values are given as medians (ranges).

1 At follow-up at Year 5, Recipients 4 and 7, and donor 8 did not complete the forms and data from Year 4 was used.

2 At Year 5, two recipients and one donor had divorced and did not complete the DAS form.

### Mood

**Figure 3.**
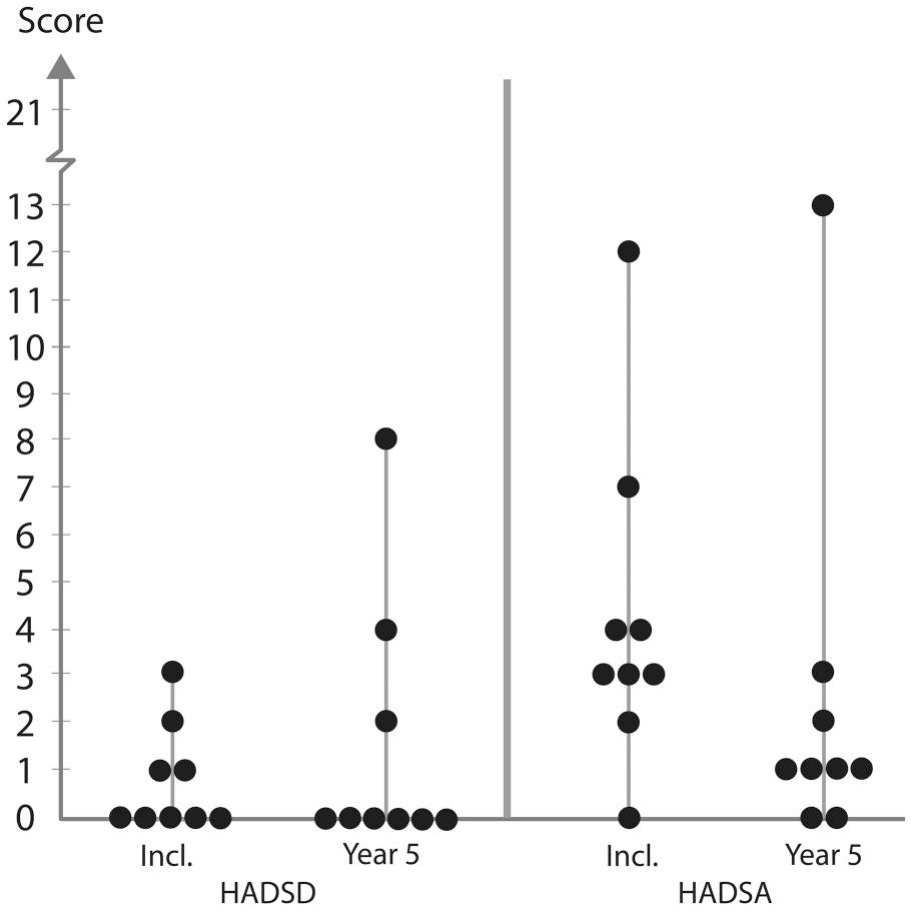
**Box plot of recipients’ individual values on depression (HADSD) and anxiety (HADSA) at inclusion and up to Year 5**.

**Figure 4.**
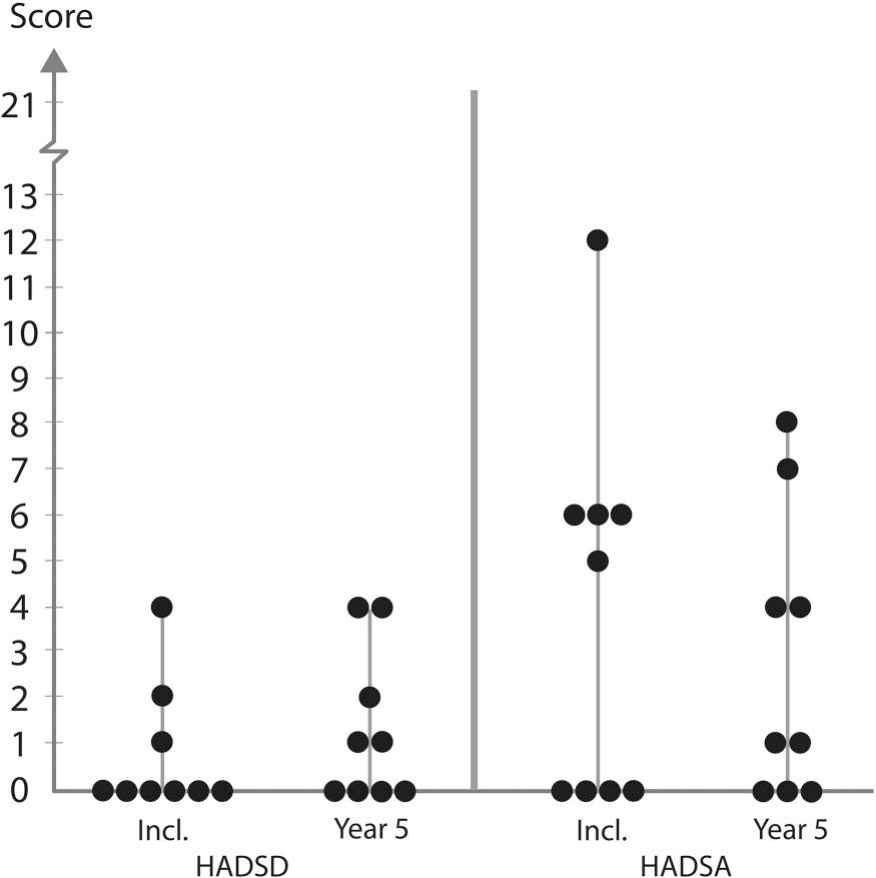
**Box plot of live donors’ individual values on depression (HADSD) and anxiety (HADSA) at inclusion (Incl.) and up to Year 5**.

The individual values and medians of HADS scores for recipients and LDs are depicted in Figs 3 and 4, respectively, with presentation of medians and ranges in Table 1.

Scores for depression (HADSD) of recipients were all low (well below 8/clinical threshold for further assessment) at the 5-year follow-up and generally similar to inclusion values. A comparable pattern was demonstrated for anxiety (HADSA) of recipients, with the exception of one value at inclusion and one value at the 5-year follow-up. Notably these two values did not represent the same recipient.

The scores of LDs regarding depression (HADSD) and anxiety (HADSA) showed comparable values as for the recipients, with two HADSA exceptions, one at inclusion and one at the 5-year follow-up. These two values were reported from different LDs. The LD who had elevated score at inclusion reported high anxiety, possibly related to upcoming surgery, and the LD with an elevated score at Year 5 had several major changes in life circumstances before that time point, and these events were not related to the UTx.

### Relationship

The median scores for marital satisfaction (DAS) are shown in Table 1. In general, recipients and LDs stated high marital satisfaction at inclusion and at the 5-year follow-up. Two recipients and one LD divorced during the study period. Their last scores before divorce were 115, 118, and 130, respectively. Thereafter they conducted no further assessment for DAS.

## Discussion

UTx, as an infertility treatment for AUFI, is undergoing transition from an experimental stage to a clinical phase. This new type of infertility treatment is being continuously evaluated regarding medical and reproductive outcomes. However, equally important components to assess are psychosocial aspects of the patients involved. Such assessments must be long-term and include evaluations of the different participants of a UTx procedure, in LD UTx including the donor as well as the recipient. In the present first ever 5-year follow-up of a UTx cohort, the results concerning health-related quality-of-life and mental well-being of recipients and LDs showed a general stability but with some exceptions, possibly related to negative reproductive outcomes and LD age. At this time, all but one recipient had undergone hysterectomy, which is the definite reproductive endpoint of the UTx procedure. To have undergone the procedure of recipient hysterectomy is an important stage to fully understand the impacts of UTx on mental well-being and health-related quality-of-life in both recipients and LDs.

It is well known that undesirable outcomes of fertility treatments affect psychological well-being in a negative way, as covered in a review by Gameiro and Finnigan (2017). This general pattern of a relationship between reproductive outcome and psychological health was also seen among the specific recipient of the present study, who had not achieved a live birth. Notably, the two recipients with early graft failures, showed a reversible decrease in psychological health and returned to normal levels at Year 5. One of the recipients with a successful uterine graft and reaching parenthood twice showed a marked variability in the MCS score, with a decline after first childbirth and a further decline during her second pregnancy. However, the levels returned to mean general population level (50) at the 5-year follow-up time. Her pattern could well be explained by the fact that the transition of becoming a parent is an evolving process with expected declines in psychological well-being during the first years of parenthood (Qu and de Vaus, 2015). Noteworthy is that the majority of recipients with live births in this study cohort did not demonstrate this pattern of decline in MCS, which further supports our notion that this is a highly selected group regarding stable psychological traits. The negative deviations in physical health-related quality-of-life of recipients do not seem to be fully associated with UTx outcomes, as seen in comparisons of recipients with early graft failure and recipients with successful outcomes with live birth. It is reassuring that the women and their respective LDs who experienced early graft failures found themselves to be mentally stable after the initial period of grief. Since graft failure always will be a possible complication of UTx, the knowledge about long-term stability is of use when counselling future recipients and LDs who experience graft failure outcomes. However, repeated implantation failures and miscarriages, without achieving live birth, will understandably have negative effects on psychological health. It should be noted that most of the negative deviations found in the present study were slight, but these have to be explored further to understand. Naturally, there is a lack of in-depth knowledge of this UTx group at this early stage of the field and further understanding will come with accumulation of research studies presenting quantitative and qualitative data.

There are publications indicating an increased risk of depression long-term after hysterectomy for several non-UTx reasons (Harnod *et al.*, 2018; Choi *et al.*, 2020) and it should be pointed out that both recipients and LDs in the UTx process undergo hysterectomy at some stage. Interestingly, the UTx recipient cohort is unique in regard to assessing post-hysterectomy quality-of-life since the majority is born without uteri and the common state for women with MRKHs, the most common recipient diagnosis in UTx, is a life without a uterus. Furthermore, the LDs of the present study have undergone hysterectomy on a novel and unusual indication, in comparison to women of general population and may therefore react differently. Our findings show that the recipient cohort was back to baseline values (below 8) in HADSD at 5 years, except for one recipient who scored 8. Even considering this, the LDs were all scoring low at baseline and at the last follow-up. Importantly, this indicates a more favourable behaviour concerning depression after hysterectomy as part of the UTx process, for both recipients and LDs, than after hysterectomy for other, more common indications. This outcome can have several explanations, such as that recipients embark on UTx with the knowledge that hysterectomy is an expected step of the process. Likewise, the LDs undergo hysterectomy as a planned, surgical intervention with a very specific goal for use of the organ.

The LDs of the present study showed some deviations regarding physical health-related quality-of-life (PCS) with a possible relationship to age of LD. Continuous negative deviations in PCS were seen among the three oldest LDs of the present study, all being above 60 years of age at the uterus donation. They all experienced physical health problems associated with aging, such as pain from lower back or legs, which most likely were not associated to donor hysterectomy (Brännström *et al.*, 2022). These are specific components concerning physical abilities in normal daily life, which can be expected to be affected due to normal aging. Nevertheless, there is a possibility that serving as an LD hastens a decline in physical status and this needs to be assessed in future studies. At Year 5, no LD reported scores under normative values in mental well-being (MCS). Collectively, these 5-year results of MCS and PCS of LDs indicate that the long-duration LD hysterectomy surgery and also the event of a negative reproductive outcome of the organ donated to a close relative or family friend, do not have any long-term negative influences on the LD. It is unlikely that longer follow-ups than 5 years will be published concerning this population. The present study had ethical permission to follow the participants for this study period but not for longer and there will always be the problem of keeping track of the LDs for several years after uterus donations. Furthermore, it may not be considered adequate to associate life-concerns to one surgical procedure performed more than 5 years prior, since many other major life events may have occurred.

Based on the results of continued variations of health-related quality-of-life of recipients within the present study, with negative deviations in the MCS, possibly related to worries about graft survival, achievement of pregnancy, and stresses of parenthood in the event of live birth, we suggest that regular assessment with SF-36 is a useful and simple tool in order to detect those in need of further psychological follow-up and support. In the present study, the questionnaires were distributed yearly, but our recommendation would be that more frequent distribution during sensitive periods should be considered. However, declines in SF-36 would not be sufficient as an indicator to offer relevant psychological support and/or possible interventions. In order to do so, the data from SF-36 has to be combined with data from more specific tests concerning anxiety and depression. Regarding reported anxiety and depression (HADS), the recipients were all below the threshold for further assessment at Year 5 and this pattern of well-being mirrors the MCS scores of SF-36.

Even though two recipients and one LD divorced during the study period, these events were not preceded by deviations in DAS scores to detect decline in marital satisfaction. This finding suggests that it could be questioned whether the DAS score is useful to detect relationships in distress among women in an UTx situation. Another interpretation could be that the participants tended to answer in socially expected ways at inclusion, thereby avoiding risk for exclusion. Divorce is a relatively common marital life event, and we speculate that the divorces seen in our cohort were not UTx-related. However, we have to remain open to the fact that UTx may be a contributing factor to these events, and we suggest that the UTx-related possible strains on marital relationship should be included in consent discussions prior to UTx for LDs, recipients, and partners. Moreover, this should be covered and discussed in future ethical discussions and papers surrounding UTx. The results of the present study suggest that evaluation with questionnaires seems to be insufficient in order to gain accurate information on relationship stability and it would be beneficial to combine questionnaires with in-depth interviews and at least yearly clinical follow-ups by a clinical psychologist.

We acknowledge some limitations of the study, which are mostly related to the limited number of participants and that the study solely covers result from one UTx centre, with a homogeneity in background factors of participating women. Given the homogeneity of the cohorts of the present study, the results are not necessarily generalizable to LD UTx procedures with altruistic, non-related LDs, or to recipients after DD UTx or recipients with non-MRKHs causes of uterine infertility. A strength of the data is the uniqueness of this first and longest follow-up of recipients and LDs of UTx.

Previous studies on UTx populations have shown that receiving a uterus will affect self-image and autonomy in a positive way (Järvholm *et al.*, 2020; Wall *et al.*, 2022) and that the act of donating a uterus has the benefit of bringing a feeling of helpfulness (Warren *et al.*, 2018). However, achieving parenthood will always be the ultimate goal for the recipient and her related LD. When interpretating the overall results of the present study, it should be kept in mind that there were close relationships between LDs and recipients in all cases. This could possibly affect psychological wellbeing, in both positive and negative ways, mostly associated with whether parenthood is achieved or not. The relationship between recipient and LD can be a source of support and reduce loneliness by providing a feeling of ‘we are in this together’ but it can also become an obstacle in expressing feelings of strain to avoid worrying each other. This contrasts with non-related, altruistic donations, which could be either open or anonymous, and where personal bonds are absent. One can speculate that the health-related quality-of-life of non-directed altruistic LDs would be different from the results of the present study. For altruistic LDs, a positive feeling with their contribution to UTx would probably appear relatively close to surgery, where their donation creates the possibility for another woman’s wish for a child (Warren *et al.*, 2021). In contrast, such a group of non-directed LDs would not get the possible positive reinforcement when the recipient is pregnant and becomes a mother.

Other important aspects of UTx are that meticulous follow-up of the children born should be conducted. Up-coming studies of future UTx candidates may also come to different conclusions in relation to increased heterogeneity among recipients, such as including other conditions than MRKHs, or different circumstances regarding the financial situation when UTx moves from a research-trial setting, with most costs covered by grants of the research group, to a clinical setting with large coverage by insurance or a federal health system or entirely out-of-pocket payment by the patients. Also, cultural and religious aspects may have impacts on psychosocial well-being both for recipients and LDs. In addition, given the unique confluence of the stressful experience of infertility and fertility treatments, a growing awareness of risks for pre- and post-partum mood disorders exists for the general infertile population. Apart from that, the multiple major surgical interventions for UTx recipients and the surgical intervention for LDs, with relational complexity in the case of directed LD-UTx donation, are highly likely to influence psychosocial outcomes. Thus, the impact cannot be fully captured via standardized measures developed for use in general non-UTx populations.

The present results suggest that follow-up for 5 years after UTx should be considered sufficient, since stable levels were reached for most participants. Importantly, both recipients and LDs did not react with depression after hysterectomy, as previously studies have shown after hysterectomy for other benign indications. A future consideration is that other types of vulnerability will be detected in upcoming groups undergoing UTx, when the treatment moves from research trials to clinical care. In-depth knowledge around UTx and the patients involved is warranted to increase the awareness of the full complexity of each stage of the long period of UTx as well as to gain understanding of different subgroups to fully capture the impact of UTx and associated mental well-being. Nevertheless, to offer support when facing well known strains, such as poor outcomes or difficulties in achieving parenthood, is a very reasonable basic level of psychological support in UTx.

## Data Availability

The data underlying this article will be shared on reasonable request to the corresponding author.
